# Role of platelet-derived endothelial cell growth factor/thymidine phosphorylase in fluoropyrimidine sensitivity

**DOI:** 10.1038/sj.bjc.6600808

**Published:** 2003-03-18

**Authors:** M de Bruin, T van Capel, K Van der Born, F A Kruyt, M Fukushima, K Hoekman, H M Pinedo, G J Peters

**Affiliations:** 1 Department of Medical Oncology, VU University Medical Center, De Boelelaan 1117, 1081 HV Amsterdam, The Netherlands; 2 Taiho Pharmaceutical Co. Ltd., 1-27 Misugidai, Hanno-Shi, Saitama 357-8527, Japan

**Keywords:** thymidine phosphorylase, 5-fluorouracil, 5′-deoxyfluorouridine, ftorafur, trifluorothymidine, thymidine phosphorylase inhibitor

## Abstract

Platelet-derived endothelial cell growth factor (PD-ECGF)/thymidine phosphorylase (TP) catalyses the reversible phosphorolysis of thymidine to thymine and 2-deoxyribose-1-phosphate and is involved in the metabolism of fluoropyrimidines. It can also activate 5′-deoxyfluorouridine (5′DFUR) and possibly 5-fluorouracil (5FU) and Ftorafur (Ft), but inactivates trifluorothymidine (TFT). We studied the contribution of TP activity to the sensitivity for these fluoropyrimidines by modulating its activity and/or expression level in colon and lung cancer cells using a specific inhibitor of TP (TPI) or by overproduction of TP via stable transfection of human TP. Expression was analysed using competitive template-RT–PCR (CT-RT–PCR), Western blot and an activity assay. TP activity ranged from nondetectable to 70678 pmol h^−1^ 10^−6^ cells, in Colo320 and a TP overexpressing clone Colo320TP1, respectively. We found a good correlation between TP activity and mRNA expression (*r*=0.964, *P*<0.01) in our cell panel. To determine the role of TP in the sensitivity to 5FU, 5′DFUR, Ft and TFT, cells were cultured with the various fluoropyrimidines with or without TPI and differences in IC_50_'s were established. TPI modified 5′DFUR, increasing the IC_50_'s 2.5- to 1396-fold in WiDR and Colo320TP1, respectively. 5-Fluorouracil could be modified by inhibiting TP but to a lesser extent than 5′DFUR: IC_50_'s increased 1.9- to 14.7-fold for WiDR and Colo320TP1, respectively. There was no effect on TFT or Ft. There appears to be a threshold level of TP activity to influence the 5′DFUR and 5FU sensitivity, which is higher for 5FU. Even high levels of TP overexpression only had a moderate effect on 5FU sensitivity.

Platelet-derived endothelial cell growth factor (PD-ECGF) is an angiogenic factor discovered in the late 1980s (Miyazono *et al*, 1987; Ishikawa *et al*, 1989). Sequence analysis of the gene revealed a stretch of 120 amino acids to be identical to thymidine phosphorylase (TP), an enzyme catalysing the reversible phosphorolysis of thymidine to thymine and 2-deoxyribose-1-phosphate (dR-1-P) (Furukawa *et al*, 1992). Subsequently, this enzymatic activity was identified for PD-ECGF (Moghaddam and Bicknell, 1992; Usuki *et al*, 1992). The two enzymes are considered to be identical and are designated as TP. The protein is expressed in normal tissues and cells, including macrophages, Kupffer cells, endothelial cells, ovary, salivary gland and brain (Fox *et al*, 1995). Increased TP expression, compared to normal tissue, was found in breast (Moghaddam *et al*, 1995), bladder (O'Brien *et al*, 1995, 1996), gastric (Takebayashi *et al*, 1996a), colorectal (Takebayashi *et al*, 1996b), lung (Giatromanolaki *et al*, 1998) cancer and several other tumours in numerous histochemical studies. In general, a high TP has been shown to be a prognostic factor for poor survival in gastric and colorectal cancer (Takebayashi *et al*, 1996a, 1996b; Matsumura *et al*, 1998; van Triest *et al*, 2000), but in oesophageal carcinoma there are conflicting reports (Ikeguchi *et al*, 1999; Koide *et al*, 1999) about its prognostic significance.

Besides its angiogenic action, the enzymatic activity of TP plays a role in fluoropyrimidine sensitivity, being able to activate 5-fluorouracil (5FU) and 5′-deoxyfluorouridine (5′DFUR) (Ackland and Peters, 1999), and an increased expression was related with a better outcome of treatment with 5FU and its derivatives (Fox *et al*, 1997; Saito *et al*, 1999). The potential actions of TP in the metabolism of various fluoropyrimidines are depicted in Figure 1

**Figure 1:**
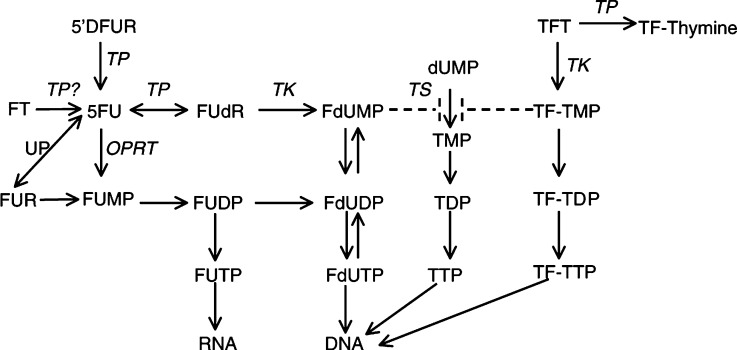
Scheme showing the possible metabolic pathways for 5FU via TP, UP (uridine phosphorylase) and orotate phosphoribosyltransferase (OPRT) and its different targets: TS inhibition via FdUMP and incorporation of FdUTP and FUTP into DNA and RNA, respectively. 5′-Deoxyfluoro-uridine is an intermediate in the conversion of Capecitabine to 5FU. Finally, the metabolic fate of TFT is shown, which can be degraded by TP or activated by TK, resulting in TS inhibition and DNA incorporation.

. TP activates 5′DFUR to 5FU by cleaving the 5-deoxyribose moiety, while by addition of 2-deoxyribose-1-phosphate TP can activate 5FU to 5-fluoro-2′-deoxyuridine, a precursor of FdUMP which inhibits thymidylate synthase, responsible for *de novo* thymidylate synthesis. Recently, there is renewed interest in the role of TP, since it activates 5′DFUR, an intermediate in Capecitabine (Xeloda) metabolism to 5FU. Capecitabine is a newly designed oral fluoropyrimidine carbamate which is converted to 5FU in three steps, the first step is catalysed by carboxyl esterase located almost exclusively in the liver, the second step by cytidine deaminase expressed in the liver and various types of tumours, and the last by TP which is higher in tumours than in normal tissues thus ensuring an enhanced efficacy (Miwa *et al*, 1998).

Trifluorothymidine (TFT) has previously been used in antiviral therapy and has been evaluated for cancer therapy as a single agent (Ansfield and Ramirez, 1971; Warrell Jr *et al*, 1979). It has shown efficacy in 5FU-resistant tumour cell lines bypassing the resistance mechanisms of these cells (Fukushima *et al*, 2000). Trifluorothymidine is inactivated by TP to trifluorothymine. This inactivation can be prevented by combining it with a specific thymidine phosphorylase inhibitor (TPI), which increased the bioavailability (Fukushima *et al*, 2000). This combination of TFT and TPI in the molar ratio 1:0.5, called TAS-102, is currently tested in phase I trials and can be administered orally (Thomas *et al*, 2002).

Also for another 5FU prodrug, Ftorafur (Ft), it has been postulated that it might be activated by TP (Kohne and Peters, 2000). Ftorafur is part of the oral formulation of UFT and S-1.

To determine to what extent TP plays a role in sensitivity to these fluoropyrimidines, we modulated TP activity in several colon cancer cell lines and one non small cell lung cancer (NSCLC) by inhibiting TP activity with TPI, and/or by overexpression of TP via stable transfection with a plasmid containing the cDNA for TP.

## MATERIALS AND METHODS

### Chemicals

Dulbecco's modified Eagle's medium (DMEM) RPMI 1640 and foetal calf serum (FCS) were obtained from Gibco BRL (Life Technology, Breda, The Netherlands). 5-Fluorouracil and 5′DFUR were purchased from Sigma Chemicals Co. (St Louis, MO, USA), Ft, TFT and TPI were provided by Taiho Pharmaceuticals (Hanno, Japan). Hybond ECL nitrocellulose membranes, Hyperfilm ECL and ECL (plus) detection kit were obtained from Amersham International (Buckinghamshire, UK). The primary polyclonal antibody was goat anti-human PD-ECGF (R&D Systems, Abingdon, UK), the secondary antibody was peroxidase-conjugated rabbit anti-goat (Dako, Glostrup, Denmark). RNAzol was obtained from Campro Scientific (Veenendaal, The Netherlands), Moloney Murine Leukemia Virus Reverse Transcriptase (M-MLV-RT) from Promega (Madison, WI, USA), deoxynucleotides (dNTPs), random hexamers and Taq polymerase from Pharmacia Biotech (Roosendaal, The Netherlands). All other chemicals were of analytical grade and commercially available.

### Cell lines and transfection

The origins of the human colon carcinoma cell lines, Lovo, WiDR, HT29, SW1369, SW948, Colo320 and of that of the human NSCLC, H460 have been described previously (Tolis *et al*, 1999; van Triest *et al*, 1999). Colo320TP1 and H460TP2 are transfected variants of Colo320 and H460. All colon cell cancer lines were maintained in DMEM supplemented with 10% FCS, H460 was maintained in RPMI with 10% FCS. All cells were cultured at 37°C in a 5% fully humidified atmosphere. Cell lines were growing exponentially as monolayers during the course of all experiments.

Colo320 and H460 cells were transfected with TP. The pBABE puromycin vector containing human TP was a kind gift from Professor IJ Stratford (School of Pharmacy and Pharmaceutical Science, University of Manchester, UK) (Jones *et al*, 2002). Although the vector is designed for viral transfection, we used it for direct transfection without packaging the DNA. Cells were transfected with 10 *μ*g of vector using Superfect (Qiagen, Crawley, UK), according to the manufacturer's protocol. Selection was made using increasing concentrations of puromycin (ICN Biomedicals, Aurora, OH, USA). Independent clones were selected and tested for expression of TP by Western blotting. After selection, the clones were maintained in 1.5 *μ*g ml^−1^ of puromycin and were passed once without puromycin before each experiment.

### Western blot analysis

For determining TP expression, logarithmic growing cells were harvested and cell pellets were lysed by lysis buffer (1% Triton X-100; 150 mM Tris-HCL, pH 7.6; 5 mM EDTA), sonificated, and centrifuged, for 10 min 14 000 *g* at 4°C. Protein content of each sample was assayed using the Biorad assay (BioRad Laboratories, Richmond, CA, USA). Thirty micrograms protein of each sample was loaded, separated on a 12.5% SDS–PAGE gel and electroblotted onto a nitrocellulose membrane. Membranes were incubated overnight at room temperature in blocking buffer: 1% bovine serum albumin (BSA; Boehringer Mannheim, Germany), 1% milkpowder; TBS-T (10 mM Tris-HCl pH 8.0, 0.15 M NaCl; 0.05% Tween-20) to prevent aspecific antibody binding. After blocking, the membranes were incubated with the primary antibody goat anti-human PD-ECGF (1/1000), followed by horseradish peroxidase-conjugated rabbit anti-goat antibody (1/2000). Enhanced chemoluminescence (ECL plus) was used for detection, and protein expression was quantified by densitometric scanning (model GS-690 and Molecular analist, BioRad Laboratories, Richmond, CA, USA). Recombinant PD-ECGF (R&D systems, Abingdon, UK) was used as a control in a dilution allowing optimal quantification in a linear range.

### Competitive template RT–PCR to determine TP mRNA expression levels

The quantitative RT–PCR technique is based on the coamplification of a competitive template (CT) designed specifically for each different target. The principles have been described in detail elsewhere (Willey *et al*, 1998; Rots *et al*, 2000; Crawford *et al*, 2001).

RNA was extracted from 5 × 10^6^ cells by the RNAzol™ method, checked for DNA contamination and reverse transcribed by random hexamers as described by the manufacturer with minimal modifications (Rots *et al*, 2000). Competitive templates were designed for *β*-actin (Rots *et al*, 2000) and TP, using the primer sets shown in Table 1

**Table 1:** Primers for RT-CT-PCR of TP to synthesise competitive templates (F and CT) and to coamplify CT and native template (NT) (F and R)

**Target**	**Position**	**5′→3′ sequence**	**Genbank**
PD-ECGF/TP	F 622	ATC CAG AGC CCA GAG CAG ATG C	M63193
	R 1045	TGG TGA CCA GGT CCC TTA AGT CTG	
	CT 893	R+CCA ACC AGC GTC TTT GCC AG	

F is the forward primer, R is the reverse primer, CT is the primer used to generate the CT.

. Competitive templates were produced from a cell line known to contain a considerable amount of TP activity (Colo320TP1). Competitive templates were dissolved in standardised solutions. Polymerase chain reaction was used for coamplification of the cDNA samples with CTs to ensure accurate quantification of the native target (NT). In order to normalise TP expression to that of *β*-actin, one single master mix was prepared for every cDNA sample containing PCR buffer (1 ×), dNTPs (200 *μ*M), Taq polymerase (0.02 U *μ*l^−1^), sample cDNA (1–3 *μ*l) and the appropriate CT mix (1–3 *μ*l) in a volume of 49 *μ*l. One microlitre of premixed primers (0.05 *μ*g *μ*l^−1^) of TP and *β*-actin primers were added to aliquots of the master mix and reaction mixtures were overlaid with 50 *μ*l of mineral oil. cDNA samples were amplified in a MJ Research PTC-2000 apparatus (Biozym, Landgraaf, the Netherlands) with 1 min steps of denaturation at 94°C, primer annealing at 58°C and elongation at 72°C for 35 cycles starting with a hot start at 94°C. PCR products were separated by 120 V electrophoresis for 2 h on 2% agarose gel containing 0.1 mg ml^−1^ ethidium bromide. The intensity of the NT and CT bands was quantified by digital image analysis using Scion Image (NIH, Bethesda, DC, USA). Concentrations of NT molecules of TP and *β*-actin in the cDNA samples were calculated by the ratio of NT/CT after amplification and the molarity of the CT mixture used as described previously (Rots *et al*, 2000). The relative expression of TP mRNA was given as the ratio of the concentration NT of TP *vs* NT of *β*-actin.

### Thymidine phosphorylase activity

The TP activity was determined using an assay previously described (Laurensse *et al*, 1988). Activity was measured using thymidine as a substrate by calculating its conversion to thymine. Depending on the TP activity, 30 or 60 × 10^6^ cells ml^−1^ 50 mM Tris/1 mM EDTA (pH 7.4) were used, which were sonificated and centrifuged at 21 000 **g** at 4°C. Fifty microlitres of 21 000 **g** supernatant was mixed with 10 *μ*l 0.8 M K_2_HPO_4_, 10 *μ*l 5 mM thymidine and 130 *μ*l TRIS/EDTA (pH 7.4) buffer, and incubated for 15, 30 or 60 min at 37°C. Thymidine phosphorylase inhibitor was used at a final concentration of 10 *μ*M. The reaction was stopped by the addition of 50 *μ*l 40% trichloroacetic acid (TCA), neutralised and analysed by HPLC as described previously (Laurensse *et al*, 1988; van Triest *et al*, 2000).

### Growth inhibition experiments

To study the role of TP in fluoropyrimidine sensitivity, the sulphorhodamine B (SRB, Sigma Chemicals, St Louis, MO, USA) staining method was used (Skehan *et al*, 1990; Keepers *et al*, 1991). It has been shown by us and others that this assay produces similar results as a clonogenic assay (Perez *et al*, 1993) and is an excellent method to measure growth inhibition of anticancer drugs. Briefly, cells were seeded at densities varying from 4000 to 15 000 cells well^−1^, depending on the doubling time, ensuring exponential growth during the experiment, with or without 10 *μ*M TPI. Drugs were added after 24 h at various concentrations and cells were incubated for 72 h. Thereafter, cells were fixed with TCA, final concentration 10% and stained with SRB (0.4% wt/vol In 1% acetic acid). Optical densities were measured on a Spectra Fluor (Tecan, Salzburg, Austria) at an absorbance of 540 nm. Growth percentage was calculated as described previously, by setting absorbance of control cells after 72 h at 100% and absorbance at the time of drug addition at 0%. Values were expressed as the concentration that corresponded to a cellular growth reduction of 50% (IC_50_) when compared to the value of the untreated control cells. The IC_50_'s are represented as means and standard error of at least three values. The term dose modifying factor (DMF) is used to express the effect of TPI and is calculated by (IC_50_+TPI)/IC_50_.

### Statistics

The one-tailed paired Student's *t*-test was used to study the effect of TPI on IC_50_'s of the different fluoropyrimidines. For the correlations, the nonparametric Spearman's *ρ*(*r*) was calculated. In some cases when specifically indicated, we also used the parametric Pearson's correlation test. Changes and correlations were considered significant when *P*<0.05.

## RESULTS

### Transfection

**Figure 2:**
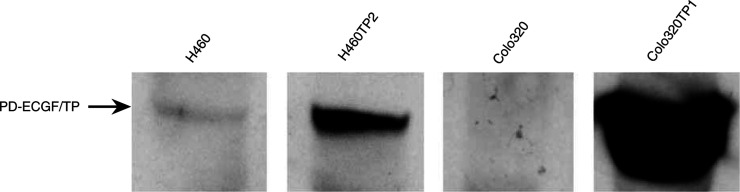
Western blot showing the TP expression of the H460 and Colo320 parental cells with their transfected counterparts H460TP2 and Colo320TP1, respectively.

Colo320 and H460 cells were transfected with full-length human TP cDNA. After selection in puromycin, several clones of both cell lines were tested for TP expression by Western blotting (Figure 2

). One high overexpressing clone of each cell line was selected for further experiments, Colo320 clone number 1 (Colo320TP1) and H460 clone number 2 (H460TP2). The clones had similar doubling times compared to the parental cell lines (data not shown).

### Thymidine phosphorylase protein, activity and mRNA expression

**Figure 3:**
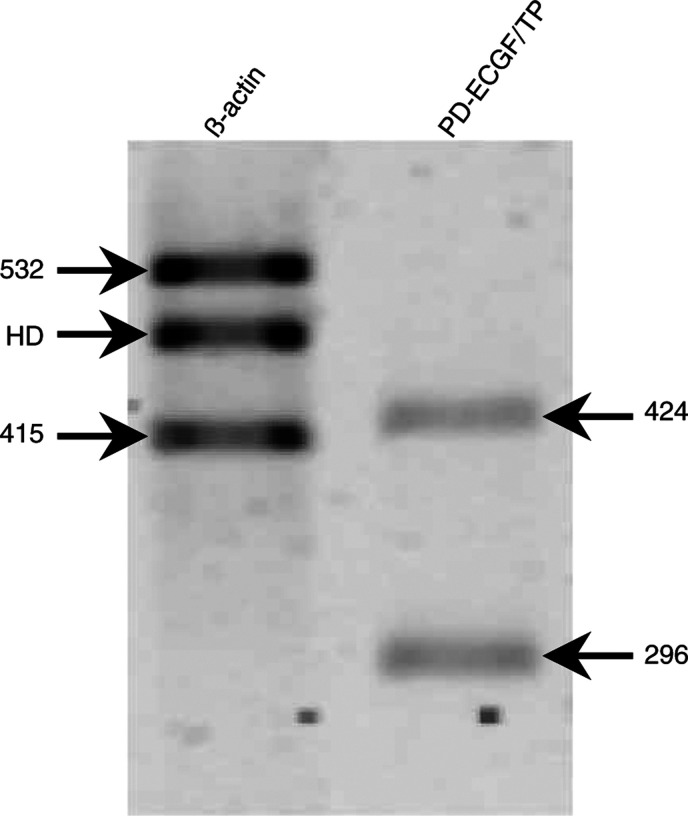
Representative example of an agarose gel on which PCR products are separated according to their expected sizes. The gels show three bands for *β*-actin: bands of 532 and 415 bp are encoded by the forward and reverse primer for the native cDNA and CT, respectively, the third band is the heteroduplex consisting of native cDNA and CT. For TP, only two bands are visible, the native cDNA of 424 and 294 bp for the CT, the heteroduplex was formed occasionally. The bands were scanned and the OD was used to calculate a ratio between the native cDNA and CT. The contribution of the heteroduplex was calculated as described previously (Willey *et al*, 1998; Rots *et al*, 2000).

In order to determine the correlation between TP activity and protein and mRNA expression, these parameters were determined with the described activity assay, Western blot and CT-RT–PCR, respectively. An example of an agarose gel with the PCR products and their expected size is shown in Figure 3

**Figure 4:**
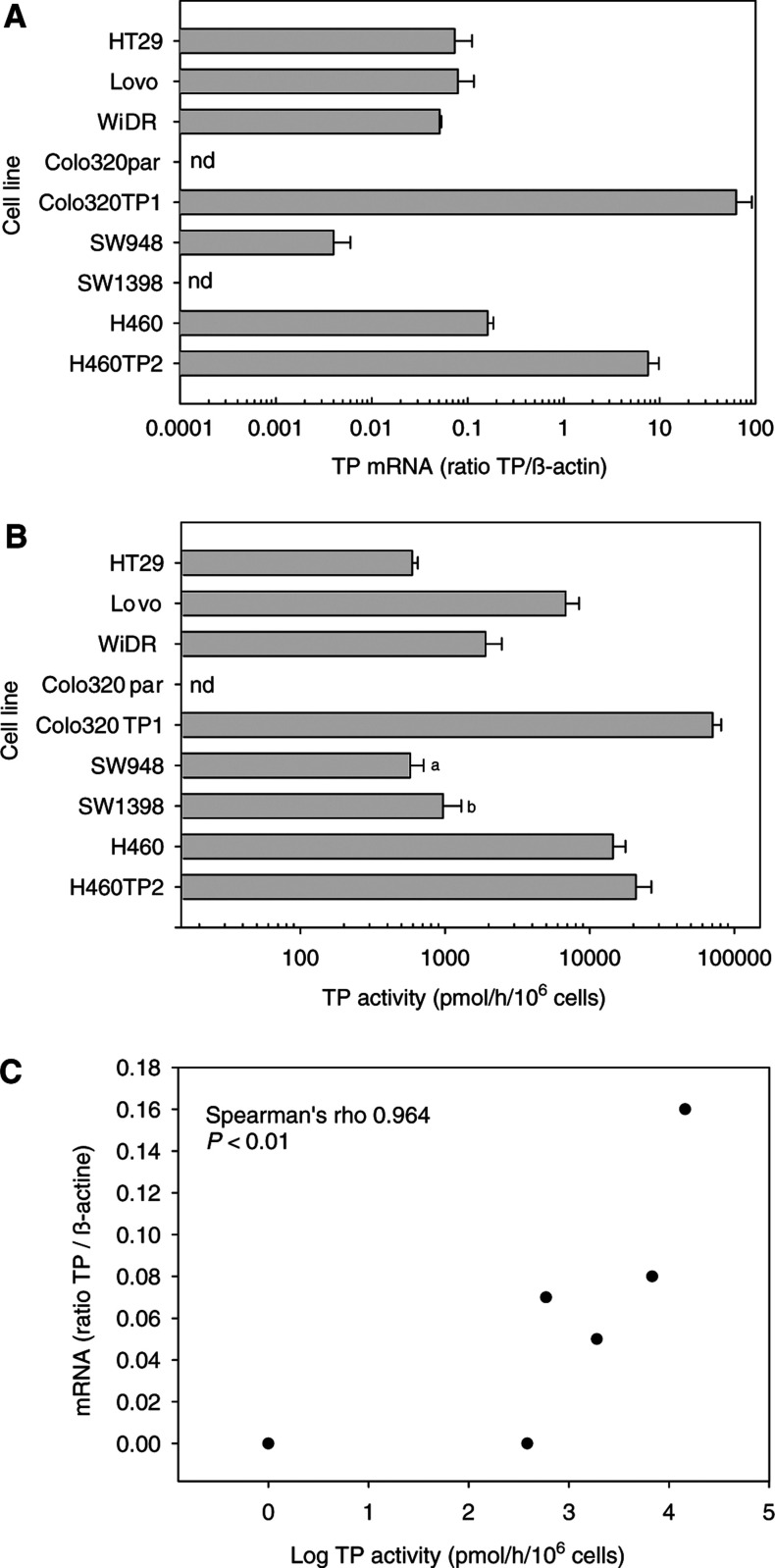
(**A**) Thymidine phosphorylase mRNA expression in the different cells lines. (**B**) Total TP activity of the different cell lines. Thymidine phosphorylase activity in SW948 cells could only be inhibited for 34% by TPI (a), while TP activity of SW1398 cells could not be inhibited by TPI (b). Thymidine phosphorylase inhibitor completely inhibited TP in the other cell lines. Protein content of the different cell lines varied from 79 *μ*g for Lovo to 194 *μ*g protein 10^6^ cells^−1^ for SW1398. (**C**) Correlation plot of TP activity and mRNA expression of the nontransfected cell lines only. There was a strong positive correlation between the two parameters. The Pearson's linear correlation coefficient was *r*=0.79 (*P*<0.05) nd, nondetectable.

. The mRNA expression results are depicted in Figure 4A

. Thymidine phosphorylase activity varied considerably among the cell lines (Figure 4B), with a moderate activity in HT29, WiDR and Lovo cells. In all cell lines, the thymidine phosphorolysis could be completely inhibited by TPI except in SW 948, in which inhibition was only 34% while this activity could not be inhibited for SW1398 by TPI. Unless otherwise stated, the measured phosphorolytic activity of the SW cell lines was adjusted according to the inhibition percentage by TPI, and this adjusted activity was used in the calculations. The H460 cell line had the highest activity of the nontransfected cells. Colo320 cells had no detectable activity but the stable transfected derivative Colo320TP1 had the highest activity (70678 pmol hr^−1^ 10^6^ cells^−1^). Figure 4C shows a correlation plot between TP mRNA and TP activity for the nontransfected cell lines only. The mRNA expression was significantly correlated with TP activity. There was also a significant correlation between mRNA expression and TP protein expression, as determined by Western blot (*r*=0.78, *P*<0.05), and protein expression also correlated significantly with TP activity (*r*=0.75, *P*<0.05) in non-transfected cell lines. Comparable protein expression patterns (data not shown) were found using a commercially available ELISA (Roche, Almere, The Netherlands).

### Fluoropyrimidine sensitivity in relation to TP levels

To determine the role of TP in the activation of 5FU, 5′DFUR, FT and the inactivation of TFT, we determined the effect of TPI on drug-induced growth inhibition (Table 2

**Table 2:** IC_50_'s of the fluoropyrimidines (expressed in *μ*M) in the presence or absence of TPI, for the different cell lines

**Cell line**	**5FU**	**5FU +TPI**	**DMF**	**5′DFUR**	**5′DFUR +TPI**	**DMF**	**TFT**	**TFT +TPI**	**DMF**	**FT**	**FT+TPI**	**DMF**
HT29	5.4±1.3	6.6±0.2	1.2	176.9±27.3	275.8±15.9	1.6	3.9±1.0	3.7±0.7	0.9	201.1±37.9	283.2±81.3	1.4
Lovo	1.1±0.1	2.5±0.2	2.2^b^	24.9±5.1	103.5±17.3	4.2^a^	0.5±0.1	0.4 ±.0.1	1.0	97.2±15.5	86±24.8	0.9
WiDR	2.5±0.5	4.7±1.0	1.9^b^	90.1±12.0	222.5±25.0	2.5^b^	2.5±0.8	3.5±1.0	1.4	203.9±44.9	236.1±27.8	1.2
Colo320	2.3±0.2	2.5±0.2	1.1	61.7±5.8	65.0±1.1	1.1	0.4±0.1	0.4±0.1	0.9	119.4±7.2	141.7±11.2	1.2
Colo320TP1	0.2±0.1	2.8±0.3	14.7^c^	0.06±0.02	81.0±10.5	1396.6^b^	0.5±0.04	0.6±0.1	1.1	ND	ND	—
SW948	3.1±1.0	4.0±1.7	1.3	208.9±7.6	255.6±14.3	1.2	ND	ND	—	ND	ND	—
SW1398	2.0±0.6	3.2±0.6	1.6	134.8±32.9	202.0±37.4	1.5	ND	ND	—	275±39.7	191.4±22.1	0.7
H460	2.0±0.2	8.6±0.4	4.3^c^	10.9±1.7	332.9±13.4	30.5^c^	0.6±0.1	0.6±0.03	0.9	ND	ND	—
H460TP2	1.6±0.2	11.1±1.0	6.8^c^	4.2±0.5	275.0±16.1	65.1^c^	0.7±0.04	0.5±0.03	0.8	ND	ND	—

Significant differences between drug and drug with TPI (Student *t*-test):

a *P*<0.05

b *P*<0.01

c *P*<0.001

Thymidine phosphorylase inhibitor was present in a final concentration of 10 *μ*M, 24 h before drugs were added. DMF is the dose-modifying factor calculated as (IC_50_+TPI)/IC_50_.

ND=not done.

). The IC_50_s for 5FU ranged from 0.2 *μ*M for Colo320TP1 to 5.4 *μ*M for HT29, and had an inverse correlation with TPI inhibitable activity (*r*=–0.63, *P*<0.05). When overall phosphorolytic activity was used, *r*=–0.78 (*P*<0.01). This high correlation, however, was mainly because of the contribution of the transfected cells; without these cells no significant correlation between TP activity and 5FU sensitivity was observed (*r*=−0.28). The transfected Colo320TP1 cell line had an IC_50_ of 0.2 *μ*M for 5FU compared to 2.3 *μ*M for the parental cell line resulting in a relative sensitivity to 5FU of 11.5. IC_50_ of 5FU was returned to parental level, 2.8 *μ*M, when TP activity was inhibited by TPI resulting in a DMF of 14.7 for the transfected cell line.

A similar picture emerged for 5′DFUR, with IC_50_'s ranging from 0.058 *μ*M for Colo320TP1 to 208.9 *μ*M for SW1398. The sensitivity to 5′DFUR inversely correlated with the TPI inhibitable TP activity (*r*=−0.80, *P*<0.01). For the overall phosphorolytic activity, *r*=−0.83 (*P*<0.01). Wild-type Colo320 was, despite lacking measurable TP activity, sensitive to 5′DFUR, with an IC_50_ of 61.7 *μ*M, comparable to WiDR, 90.1 *μ*M. This might indicate that there is another route of 5′DFUR activation in Colo320 cells, such as another phosphorylase (Peters *et al*, 1989). There was no effect of TPI on the IC_50_'s for 5′DFUR in SW948 and SW1398 cell lines, corresponding with the low and lack of inhibition of phosphoro-lytic activity by TPI. The IC_50_'s were higher for 5′DFUR than for 5FU. A good correlation between 5FU and 5′DFUR sensitivity would indicate that they would be activated by the same enzyme and would act on the same target(s). We observed a good correlation between all the IC_50_'s of 5FU and 5′DFUR (*r*=0.85, *P*<0.01), which is a reflection of a partly similar mechanism of activation.

Cell lines were sensitive to TFT with IC_50_'s below 1 *μ*M, except WiDR and HT29 which have an IC_50_ of 2.5 and 3.9 *μ*M, respectively. Trifluorothymidine seems to be the most potent of the four tested fluoropyrimidines. In this setting of 72 h continuous exposure, the sensitivity to TFT was not related to TP activity, which was expected because TP can inactivate TFT.

The sensitivity for FT, the 5FU prodrug, ranged from 97.2 to 275 *μ*M for Lovo and SW 1398, respectively. There was no significant effect of TPI on the Ft sensitivity of the nontransfected colon cancer cells of the panel. There was no correlation between IC_50_'s of Ft and 5FU.

**Figure 5:**
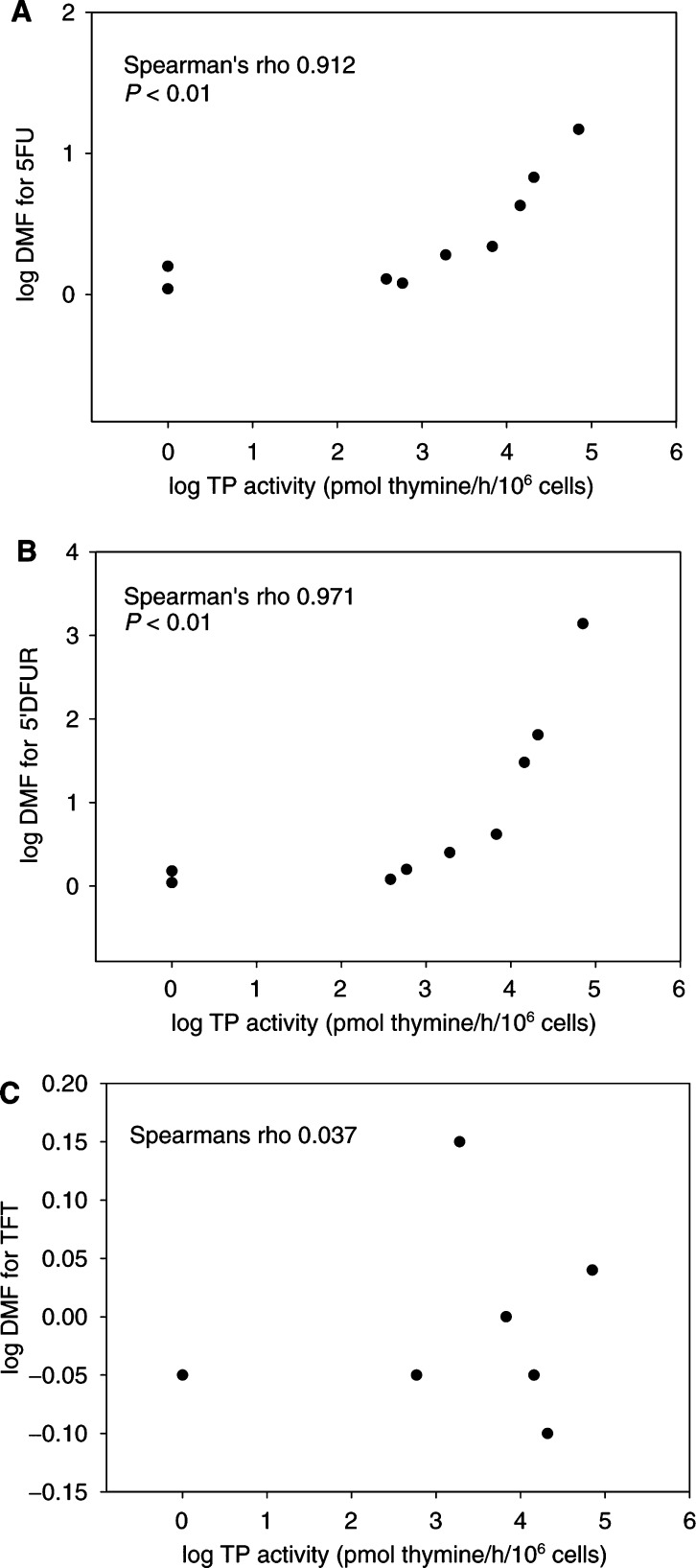
Correlation plots of TPI inhibitable TP activity of all cell lines with (**A**) DMF of 5FU, (**B**) DMF 5′DFUR and (**C**) DMF TFT.

Thymidine phosphorylase inhibitor significantly increased IC_50_'s for 5FU in WiDR, Lovo, H460, H460TP2 and Colo320TP1. The increase expressed as the DMF (ranging from 1.9 for WiDR to 14.7 for Colo320TP1), correlated with TP activity (*r*=0.91, *P*<0.01) (Figure 5

). TPI also significantly increased the IC_50_'s for 5′DFUR in WiDR, Lovo, H460, H460TP2 and Colo320TP1 by adding TPI. The corresponding DMFs correlated with TP activity of the cells (*r*=0.97, *P*<0.01), ranging from 2.5 for WiDR to 1396 for Colo320TP1.

Expression of TP mRNA also correlated with DMFs for 5FU and 5′DFUR (*r*=0.86 and 0.95, *P*<0.01, respectively), but not for the DMFs of TFT or Ft. Omission of the transfected cell lines resulted in *r*=0.70 (*P*<0.05) and *r*=0.90 (*P*<0.01) for 5FU and 5′DFUR, respectively. Thymidine phosphorylase protein levels correlated with DMF for 5′DFUR (*r*=0.70, *P*<0.05), but did not with DMFs for 5FU most likely because of the more accurate and sensitive nature of CT-RT–PCR compared with the Western blot.

## DISCUSSION

In this study, we investigated the role of TP in the sensitivity to several fluoropyrimidines: the widely used chemotherapeutic agent 5FU, its prodrugs 5′DFUR, FT and TFT, a novel oral fluoro-pyrimidine. The activation of 5′DFUR was studied because it is the final intermediate in the activation of the oral fluoropyrimidine Capecitabine (Xeloda), which is postulated to be dependent upon TP.

There was a wide range of basal TP activity in our cell panel, varying from no activity for Colo320 cells to the intermediate activity of Lovo, WidR and HT29 cells to a high activity in H460 cells and the two transfectants H460TP2 and Colo320TP1. The SW1398 and SW948 cell lines also had an intermediate activity which converted TdR to thymine, but this could not be inhibited by TPI Q1or could only by 34%. This may be explained by the fact that not TP, but the closely related uridine phosphorylase (UP), catalysed this conversion. This is in contrast to the finding that transfection of MCF7 cells with the UP gene did not influence the effect of 5FU or 5′DFUR (Cuq *et al*, 2001). Since Colo320 cells are sensitive for 5′DFUR, despite the lack of detectable cleavage of TdR, there is apparently a variability of substrate specificity of UP and TP from the different cell lines. el-Kouni *et al* (1993) described that specificity of TP for substrates varied between two different organs Q2and cancers from mouse and humans. In Colo320 cells, another pyrimidine nucleoside phosphorylase may be active, that uses 5′DFUR as a substrate but for which TdR is not a substrate (Woodman *et al*, 1980; Cao *et al*, 2002).

Using TPI and transfection, sensitivity of the cells to 5FU and 5′DFUR could be modulated either by inhibiting or enhancing TP activity to different extents. It seems that TP only plays a minor role in 5FU cytotoxicity in the nontransfected cell lines with DMFs varying from 2 to 4, whereas in transfected cell lines the DMFs go up to 7 and 14. In colon cancer cell lines with naturally occurring TP activity, the contribution as concluded by TPI inhibition is relatively low. Uptake and other activation pathways such as UP and OPRT (Peters *et al*, 1989) seem much more important. Thymidine phosphorylase may play a more important role when an additional source for the substrate for the activation reaction dR-1-P is provided. For 5′DFUR, it can be concluded that the role of TP, in determining the IC_50_, is larger with DMFs varying from 2.5 to 30 for nontransfected cell lines, and varying up to 65 and 1400 in the transfected cell lines. In previous studies (Patterson *et al*, 1995; Kato *et al*, 1997; Evrard *et al*, 1999a, 1999b; Marchetti *et al*, 2001), the effect of TP on 5FU and 5′DFUR was also demonstrated, although the enhanced sensitivity of Colo320TP1 for 5′DFUR (1396-fold) was extremely high. For example, MCF7-transfected cells had an increased sensitivity, of 165-fold (Patterson *et al*, 1995), PC9-transfected cells, 153-fold (Kato *et al*, 1997), and PROb- transfected cells, 10-fold (Marchetti *et al*, 2001). In these studies, there was also increased sensitivity to 5FU but always lower than for 5′DFUR. Other studies report that after transfection the sensitivity increase for 5FU was higher than that of 5′DFUR (Evrard *et al*, 1999a, 1999b), which is possibly because of an increased availability of dR-1-P in these cells, necessary for activation of 5FU by TP. Increase in dR-1-P availability in cells greatly enhances 5FU sensitivity mediated by TP (Peters *et al*, 1987; Ciccolini *et al*, 2001). This different role of TP in 5FU and 5′DFUR cytotoxicity is because of the fact that 5′DFUR is a prodrug of 5FU and needs an extra activation step. Activation of 5′DFUR can only occur through its conversion to 5FU, but that of 5FU can be mediated by three different pathways. Thereafter, the drugs might exert a similar mechanism of action.

There was no effect on Ft in the tested nontransfected cell lines. Recent studies show that the activation of Ft is mediated by cytochrome *P*450 enzymes (Komatsu *et al*, 2000), which have a considerable but variable expression in colon cancer cell lines (Yu *et al*, 2001) which explains the lack of correlation in IC_50_'s between Ft and 5FU.

There was no effect of TPI on TFT sensitivity, which was unexpected because it has been demonstrated that TFT is a good substrate for TP (Fukushima *et al*, 2000). We expected to see a decrease of IC_50_ for TFT in the cell lines with high TP expression when given in combination with TPI. However, since the 72 h continuous exposure might be too long to detect an effect of TPI, we decreased drug exposure times to 2 h followed by a 72 h drug-free growth, but also in this setting TPI did not affect TFT sensitivity (data not shown). Possibly activation of TFT by thymidine kinase (TK) is very efficient, preventing inactivation by TP. Trifluorothymidine possibly acts by TS inhibition and DNA incorporation. However, orally administered TFT in combination with TPI (TAS-102) seems to prevent systemic degradation (e.g. liver) of TFT resulting in increased plasma levels compared to TFT alone (Fukushima *et al*, 2000).

Use of TPI might also have an indirect effect on the sensitivity of the different fluoropyrimidines; TPI can prevent TdR degradation which might rescue cytotoxicity of 5FU and 5′DFUR. Patterson *et al* (1995, 1998) indeed described that high TP can moderate thymidine dependent rescue of TS inhibited cells. This of course depends on the intracellular TdR concentration.

We found a good correlation between mRNA expression and activity, mRNA expression and protein expression, indicating that mRNA screening of tumour samples might be sufficient to characterise the TP status, requiring a low amount of material to determine TP status. However, cell lines are homogeneous, while tumours are heterogeneous with unknown amounts of tumour, stroma and infiltrating cells, which can all contain considerable amounts of TP expression (Takahashi *et al*, 1996; Giatromanolaki *et al*, 1998; Matsumura *et al*, 1998; van Triest *et al*, 2000).

In conclusion, we determined that there is a small role of TP in the cytotoxicity of 5FU, and that this role could be increased when TP expression was increased. For 5′DFUR activation, the role of TP is much more pronounced. FT sensitivity was not dependent upon TP in the tested cell lines.
